# Autologous semitendinosus tendon graft could function as a meniscal transplant

**DOI:** 10.1007/s00167-021-06606-8

**Published:** 2021-06-08

**Authors:** Erik Rönnblad, Pierre Rotzius, Karl Eriksson

**Affiliations:** 1 Capio Artro Clinic, Stockholm Sports Trauma Research Center/Karolinska Institutet, Valhallavägen 91, 114 86 Stockholm, Sweden; 2 Södersjukhuset/KISÖS, Stockholm, Sweden

**Keywords:** Meniscal transpantation, Meniscus repair, Tendon autograft

## Abstract

**Purpose:**

Meniscectomy results in poor knee function and increased risk for osteoarthritis. Meniscal allograft transplantation is not widely used due to costs and availability. The semitendinosus tendon (ST) has the potential to remodel and revascularize in an intraarticular environment, such as ACL reconstruction. The objective for this pilot study was to investigate whether the ST graft could function as a meniscal transplant.

**Methods:**

The ST was doubled and sutured with running sutures and pull-out sutures in each end. Bone tunnels were used for root anchorage and the graft was sutured with allinside, inside-out and outside-in technique. The pull-out sutures were fixed over a button. Partial weight bearing was allowed with limited range of motion in a brace for the first 6 weeks. Evaluation was assessed using clinical examination, radiology and patient reported outcome.

**Results:**

A total of seven patients have been included between January 2018 and June 2020. Six medial transplants and one lateral transplant were performed. Mean age was 29 years. Four patients had completed the 12-month follow-up. Improvements were noted for IKDC Global Score, KOOS pain subscale and Lysholm. MRI indicated that the transplant become more wedge-like with visible roots and minor protrusion.

**Conclusions:**

Even though this is primarily a technical report the follow-up data indicate that the transplant survives and adapts in shape and capabilities to an original meniscus. There were no adverse events and the patients seem to improve in terms of pain and quality of life.

## Introduction

Meniscal injuries are common, and removal of meniscal tissue has been linked to poorer knee function and a significantly increased risk of developing osteoarthritis [1, 3, 21, 27–29, 35, 39]. Meniscal repair is favorable, however, not always achievable. Different implants have been suggested to substitute a removed meniscus [37, 38]. Despite promising early results [11, 41], scaffolds are not widely used today. Meniscus allograft transplantation (MAT) has been performed for many years [24, 31, 43]. Several studies have reported relatively successful results following MAT [9, 10, 12, 15, 18–20, 23, 26, 32, 34, 40, 42] even though its chondro-protective effect remains unclear [30, 32, 33, 36]. Patient selection is crucial and sizing issues, as well as costs and availability, are hinders that have limited the widespread use of this method [4]. Furthermore, in some countries, such as Japan, allograft tissue is not widely available.

Kohn et al. [16] have reported chondroprotective effects using a patellar tendon autograft as a meniscal transplant after meniscectomy in an animal study. They also reported successful results for both healing and cartilage protection in a clinical study using part of the quadriceps tendon as meniscal autograft transplant [16, 17]. Twelve-month results were reported to be promising but no detailed data has been published.

Johnson and Feagin presented a pilot study in 2000, where tendon autografts were used as a lateral meniscal transplant [14]. No clinical improvement or preservation of the joint space was observed. However, the patients had loss of lateral joint space and profound genu valgus at the time of surgery, suggesting that the patients were rather cases for knee replacement [14]. The semitendinosus tendon has previously been transplanted as a new meniscal rim for attachment of a collagen implant with successful outcomes [2].

The semitendinosus tendon is a well-known graft that is relatively easy to harvest with low harvest site morbidity. It has biological properties with potential to remodel and revascularize in an intraarticular environment, such as in ACL reconstruction [13, 16].

The hypothesis for this study is that the semitendinosus tendon graft can function as a meniscal transplant after total or subtotal meniscectomy, and that patients receiving a neomeniscus with semitendinosus tendon experience less post meniscectomy symptoms.

## Materials and methods

The study was approved by the Regional ethical committee (Karolinska Institutet ID number: 2016/281-31/1).

Patients were assessed for eligibility using an a priori set of patient inclusion criteria: age 20–50 years, previous history of subtotal or total meniscectomy medially or laterally, no significant osteoarthritic changes on radiographs (Ahlbäck 0–1), alignment on long alignment films producing hip–knee–angle (HKA) of maximum 3 degrees increased stress in the affected compartment, post meniscectomy symptoms (i.e., medial or lateral pain accentuated with weight bearing), no smoking. Furthermore, ligamentous stability was required, and in cases of ACL insufficiency a concomitant ACL reconstruction or revision was performed.

All patients received thorough counseling regarding the surgical procedure, its experimental nature, expectations and treatment options available.

### Evaluation criteria

Successful outcome was considered improvement of knee function and quality of life for the patient according to patient reported outcome measures (PROMs). PROMs used were Global score, Knee Injury and Osteoarthritis and Outcome Score (KOOS), Lysholm score and activity score according to Tegner. Questionnaires were answered preoperatively and 3, 6, 12 and 24 months postoperatively.

Another criterion for success was maintenance of transplant integrity. This was assessed through MRI after 3, 6, 12 and 24 months. Further radiological examination was weight bearing radiographs and HKA and traditional radiography after 6 months.

Clinical assessment including range of motion (ROM), tenderness, effusion and laxity were performed at 3, 6, 12 and 24 months.

### Surgical technique

#### Arthroscopic preparation

All surgical procedures were performed by the senior author. Antibiotic prophylactics was administered using i.v. Cloxacillin® 2 g, and the graft was imbedded in a Vancomycin® swab. In cases of ACL insufficiency a reconstruction or revision was performed.

Any remaining remnants of the native meniscus were removed and the menisculocapsular junction was debrided to obtain a fresh bleeding surface. Pie-crust of the MCL was performed when needed.

#### Graft preparation

**Fig. 1:**
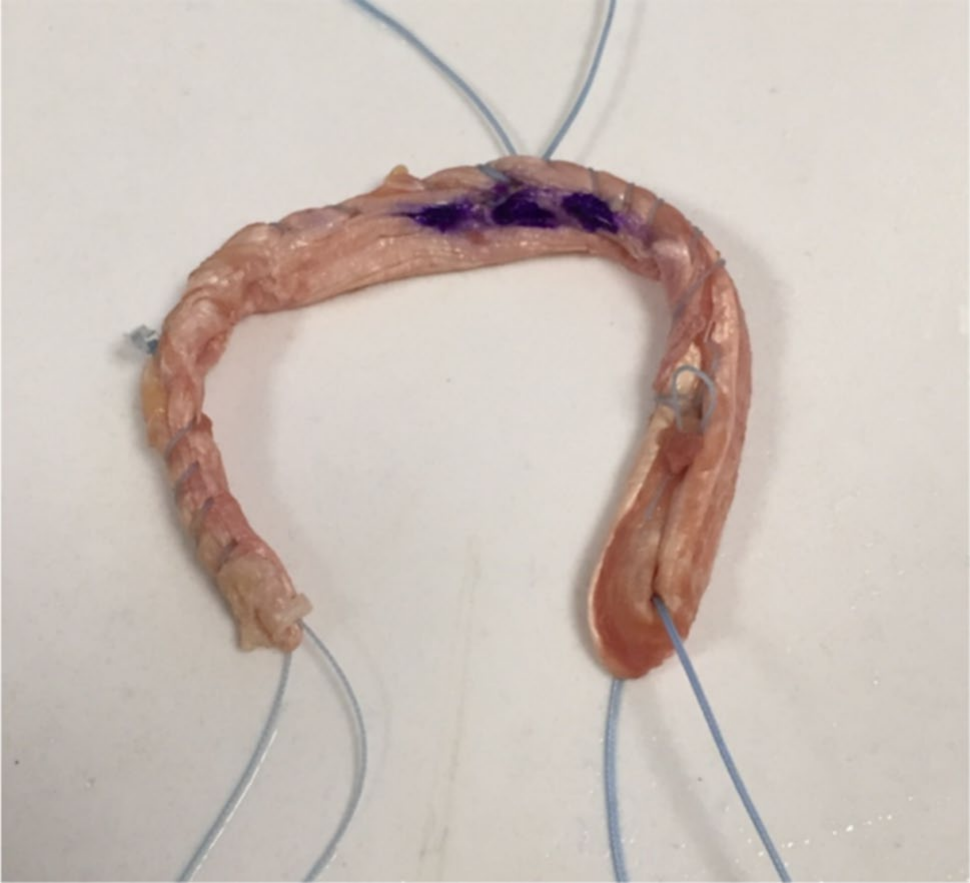
Double-folded semitendinosus tendon with sutures

After harvesting, the graft was cleaned of any muscle tissue and the flat proximal part of the tendon was folded over the distal round part creating a double-stranded loop (Fig. 1). The folded flat part of the tendon was then sutured with a running 2.0 Fiberwire® suture embedding and catching the round part. The two knots were placed in each end of the folded graft to avoid interference with the intraarticular surface. A number 2 suture-tape was used to create a Chinese finger trap of the free strands in the opposite end of the graft. The length of the grafts varied between 12 and 15 cm and diameter varied between 6 and 7 mm.

#### Tunnels

The root tunnels were created as close to the anatomic position as possible in a retrograde fashion using the meniscus root guide and flip-cutter (Arthrex®) with dimensions corresponding to the graft size.

#### Insertion and placement

**Fig. 2:**
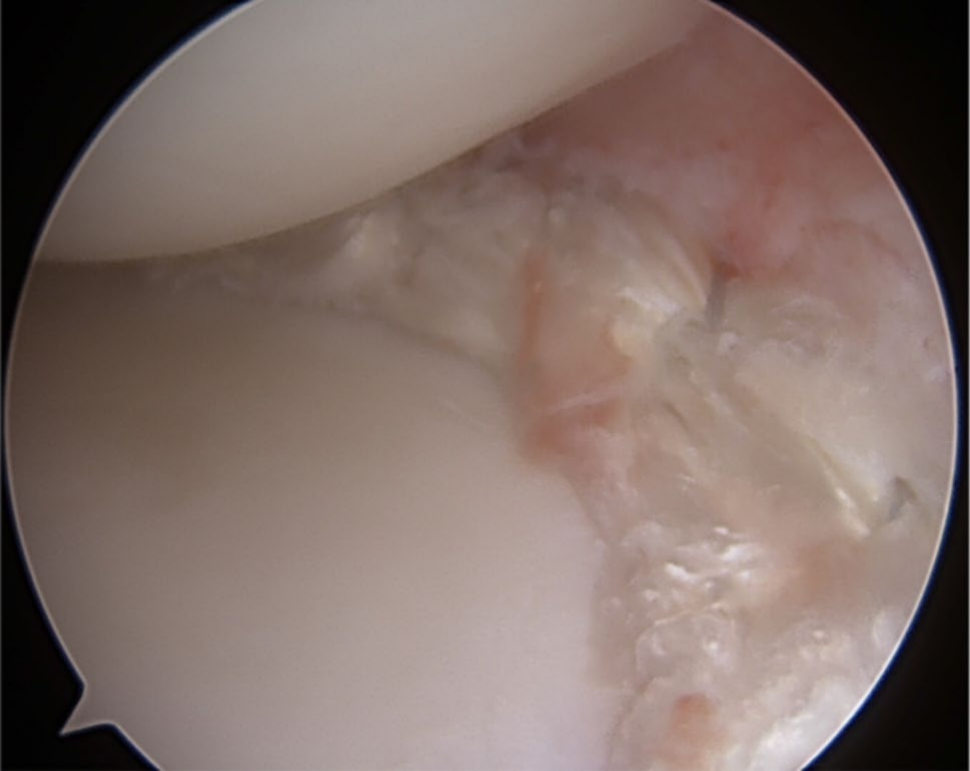
Medial meniscus transplant in position

The tendon graft was inserted through an accessory portal and the folded end of the graft was pulled into the posterior root tunnel. The graft was then pushed in place along the capsular border using a blunt instrument. The sutures from the other graft end were retrieved and pulled down the anterior root tunnel. Vertical sutures were used around the graft (Fig. 2). The inside-out and outside-in sutures were not fixed to the capsule until tension had been applied by pulling on the anterior and posterior root sutures.

### Postoperative rehabilitation protocol

**Fig. 3:**
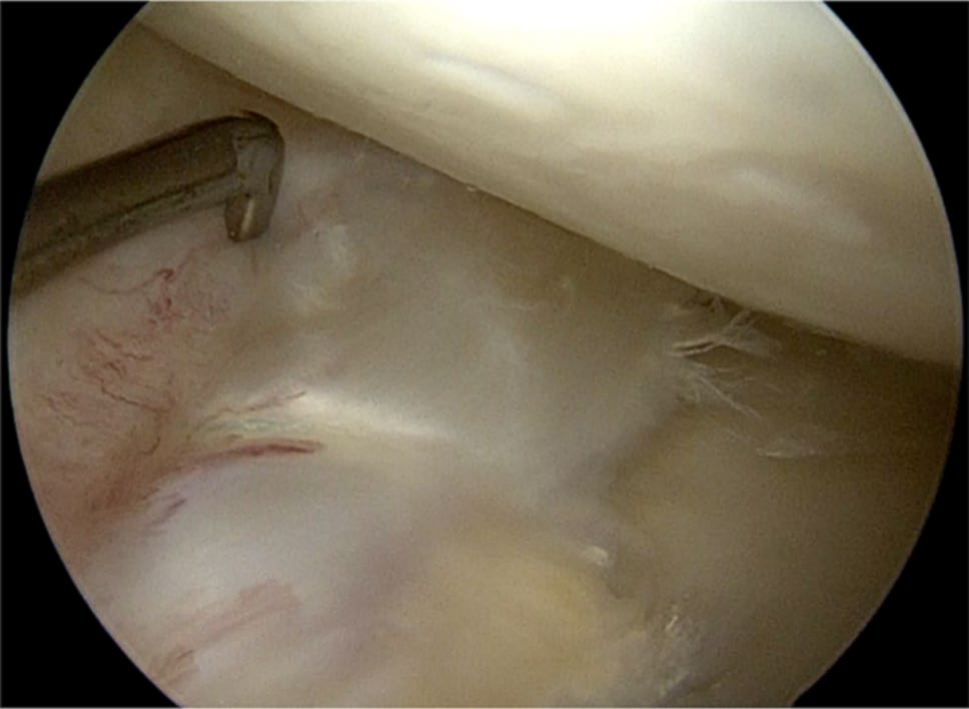
Re-arthroscopy 6-month post-operatively. Capillary ingrowth noted along the circumference of the neomeniscus

**Fig. 4:**
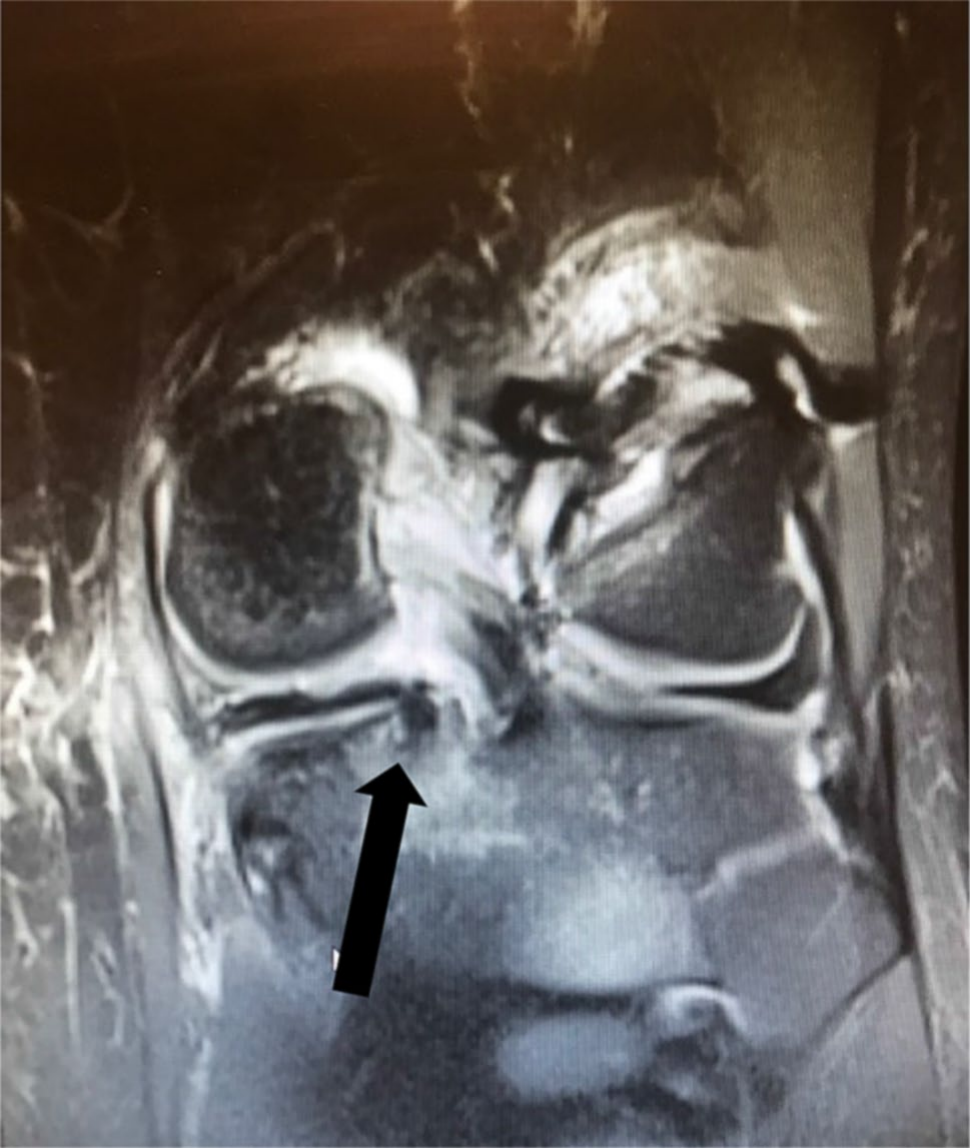
Frontal view of MRI T2-scan showing a medial meniscus transplant with the posterior root attachment (arrow)

Partial weight bearing was allowed for the first 6 weeks. A hinged knee brace was used, set at 0–30° for 3 weeks, 0–60° for 3 weeks, 0–90° for 2 weeks and unrestricted range of motion in the brace for another 4 weeks. The protocol follows the standard procedure following a suture of a sutured meniscus bucket-handle tear apart from the partial weight bearing. Weight bearing while squatting was restricted the first 4 months (Figs. 3 and 4).

### Statistical analysis

Due to the low number of patients the results are only presented in a descriptive fashion.

Statistical analyses were conducted using IBM SPSS Statistics version 23 (SPSS Inc, Armonk, New York, USA).

## Results

Seven patients were included between January 2018 and June 2020. Baseline characteristics are presented in Table 1. Results are presented on group level. No early surgical complications, such as infections or deep vein thrombosis (DVT), were registered in any of the cases.

**Table 1:** Demographic characteristics

Gender (female:male)	Age (median, range)	Side (right:left)	Meniscus (medial:lateral)	*N* of ACLR	BMI (median, range)
6:1	28 (23–41)	3:4	6:1	4	25.4 (22.1–37.2)

*ACLR* anterior cruciate ligament reconstruction; *BMI* body mass index

**Patient number two was excluded from the 12-month analysis due to failure with subtotal resection of the neomeniscus

Four patients had completed the 12-month follow-up. Global score, KOOS pain subscale, and Lysholm all increased.

### Global score

For the four patients with a retained meniscus transplant, there was an improvement in global score at 12-month follow-up (Table 2).

**Table 2:** Global score

Preop *N* = 7	3 months *N* = 7	6 months *N* = 7	12 months *N* = 4
3	3	5	6.5

Data are reported as median (range)

**Patient number two was excluded from the 12-month analysis due to failure with subtotal resection of the neomeniscus

### KOOS

The mean KOOS scores showed an improvement compared to preoperative. KOOS data is detailed in Table 3.

**Table 3:** KOOS

	Preoperative *N* = 7	3 months *N* = 7	6 months *N* = 7	12 months *N* = 4
Symptoms	45 ± 14	50 ± 14	55 ± 15	65 ± 7
Pain	51 ± 15	70 ± 5	72 ± 18	78 ± 8
ADL	62 ± 26	77 ± 14	80 ± 25	88 ± 10
Sports/Rec	20 ± 30	30 ± 32	28 ± 28	30 ± 24
QoL	16 ± 20	22 ± 12	28 ± 20	38 ± 5

Data are reported as mean ± SD

*ADL* activities in daily living; Sports/Rec, sports and recreation; *SD* standard deviation; *QoL* quality of life

### Lysholm score

There was an improvement in Lysholm at the 12-month follow-up compared to the preoperative values. Lysholm values are presented in Table 4.

**Table 4:** Lysholm score

Preoperative *N* = 7	3 months *N* = 7	6 months *N* = 7	12 months *N* = 4
41 ± 14	62 ± 10	65 ± 21	73 ± 10

Data are reported as mean ± SD

### Radiographic assessment

The signal intensity in the grafts on MRI was predominantly increased and, in some cases, slight medial protrusion was noted. The root anchoring of the graft was clearly seen in all cases. In all cases the neomeniscus presented in a wedge shaped, meniscus-like fashion.

## Discussion

The most important finding of the study was that early results indicate promising potential for the use of semitendinosus tendon as a meniscus transplant. It is important to emphasize that this paper is primarily a technical report on the use of an autologous semitendinosus tendon graft as a neomeniscus. The early follow-up data indicate that the transplant could survive, transform and remodel to a meniscus-like structure with ingrowth to the surrounding capsular tissue. As such it could potentially function as a meniscus substitution. Despite a small number of patients and as yet short follow-up period, data also indicated that most patients experienced an improvement in terms of weight bearing pain and quality of life.

### Transplant integrity

**Fig. 5:**
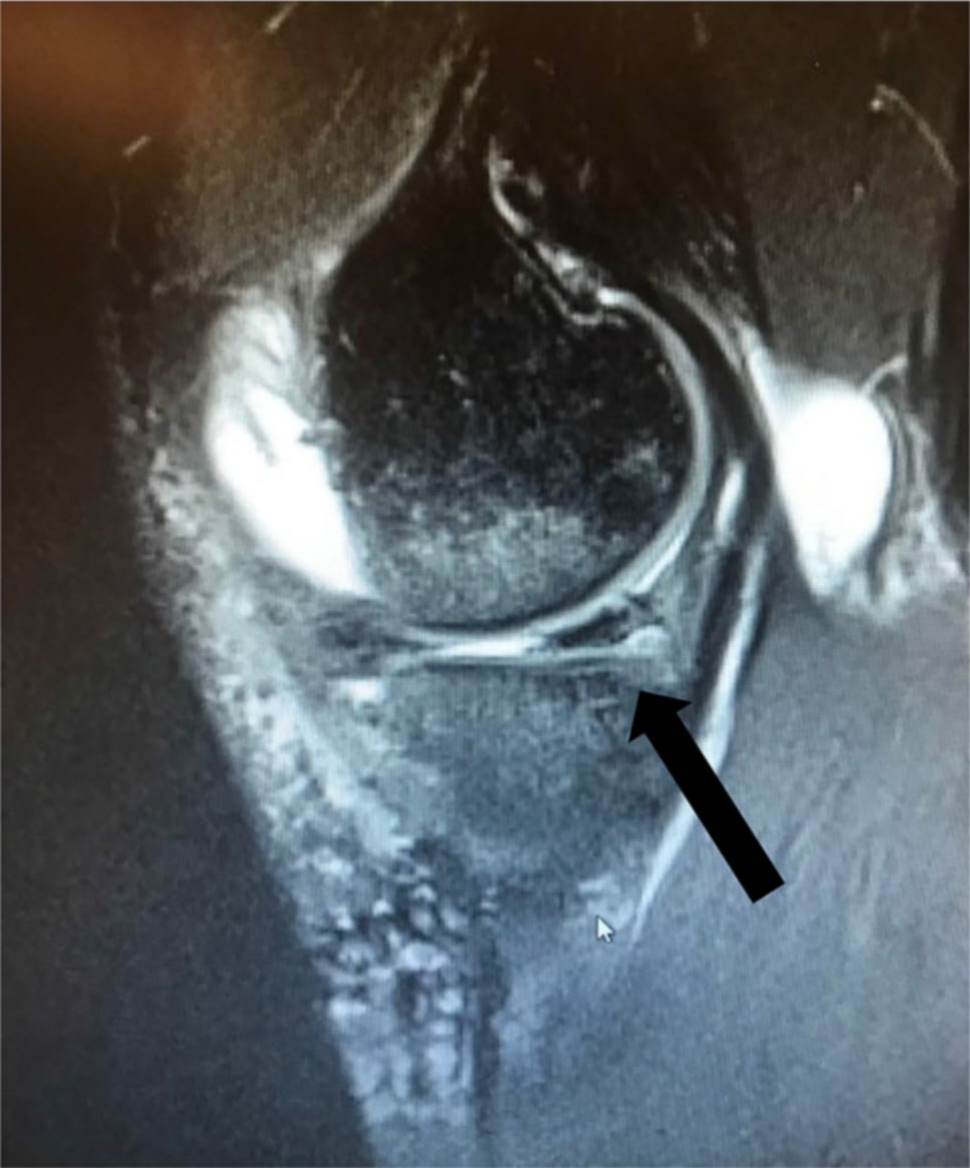
Sagittal view of MRI T2-scan showing the posterior horn of a medial meniscus transplant (arrow)

MRI scans at 12-month follow-up show signs that the transplant transforms in shape and becomes more wedge like, though with increased signal in most projections (Fig. 5). The anchorage of the roots is visible (Fig. 4). The volume of the transplant remains to be analyzed and compared to normal menisci.

One patient was excluded due to a general progression of osteoarthritis and failure of the posterior part of the transplant. In retrospect, it is plausible that the OA changes in the knee joint already seen prior to surgery were too severe to justify inclusion in the study [22].

### Surgical procedure

The surgical procedure is challenging as is all meniscal transplantations. With the extensive previous experience and knowledge in the use of the semitendinosus as an ACL graft [6–8], harvesting and preparation is well known. With the increasing awareness of meniscal root tears [5], tunnel positioning and drilling for meniscus roots has become a more common procedure.

### Patient outcome

All four patients who had completed the 12-month follow-up reported improvement in Lysholm, KOOS symptoms subscale and Global score. This is of course a small material and even though the changes are statistically significant one can question its clinical relevance. In light of this technical report, it is, however, of importance to note that the patient’s knee function did not deteriorate following surgery. No major complications were noted. Thus in the short-term perspective the safety of the procedure appears to be acceptable.

Johnson and Feagin reported non-favorable results after autograft tendon grafts as meniscus transplants [14]. Their procedures can, however, be considered salvage maneuvers as it would appear the patients already had joint changes that required a total knee joint replacement, which can explain why the results were not successful. When the OA development is too advanced, a meniscus transplant is unlikely to stop the progression, which is similar to our case no 2 (subsequently excluded from further follow-up), where the posterior part of the graft was removed at 12 months due to lack of ingrowth, graft instability and progression of OA [20].

However, with suitable intraarticular cartilage conditions a soft tissue graft could potentially serve well as a meniscus substitution as shown in the other cases in the present study. This is also in line with a recent case study that presented results from two cases, where the peroneus longus tendon was used as a meniscus transplant [25].

This study has several limitations. First and foremost the follow-up time is short, and the cohort is limited in size. It is difficult to draw any major conclusions on graft integrity and patient reported knee function on four patients after 1 year. One patient had completed the 24-month follow-up with still promising results. To be able to draw a sounder conclusion, more patients need to be included with a longer follow-up time.

Another limitation is that inclusion was not restricted to a certain BMI level. One patient included in the study had BMI > 35 which can possibly affect the outcome of that patients transplant and development of OA.

The patients in this study were not offered a MAT as alternative treatment. This is due to the low use of this method in our country as a result of logistical difficulties, costs and tradition. For further analysis it would be of interest to compare the outcome of this procedure with MAT.

With this limited number of cases it is impossible to determine whether this method is best used for medial or lateral meniscus transplantations.

## Conclusion

The use of autologous semitendinosus tendon as meniscus transplant seems to be a possible alternative to the methods used today. The patients included so far present improvement in weight bearing pain and quality of life.
